# Evaluating a deep learning AI algorithm for detecting residual prostate cancer on MRI after focal therapy

**DOI:** 10.1002/bco2.373

**Published:** 2024-05-12

**Authors:** David G. Gelikman, Stephanie A. Harmon, Alexander P. Kenigsberg, Yan Mee Law, Enis C. Yilmaz, Maria J. Merino, Bradford J. Wood, Peter L. Choyke, Peter A. Pinto, Baris Turkbey

**Affiliations:** ^1^ Molecular Imaging Branch, National Cancer Institute National Institutes of Health Bethesda Maryland USA; ^2^ Urologic Oncology Branch, National Cancer Institute National Institutes of Health Bethesda Maryland USA; ^3^ Department of Radiology Singapore General Hospital Singapore; ^4^ Laboratory of Pathology, National Cancer Institute National Institutes of Health Bethesda Maryland USA; ^5^ Center for Interventional Oncology, National Cancer Institute National Institutes of Health Bethesda Maryland USA; ^6^ Department of Radiology, Clinical Center National Institutes of Health Bethesda Maryland USA

**Keywords:** artificial intelligence, focal therapy, prostate cancer

Advancements in artificial intelligence (AI) have shown promise in standardizing medical imaging evaluations, particularly in detecting prostate cancer (PCa) on MRI.^1^ Though MRI‐based AI algorithms have been developed to detect PCa in untreated glands,^2^, ^3^ little research exists on the efficacy of such models after prostate ablation. While focal therapy (FT) targets and destroys localized PCa, it usually distorts prostate anatomy, making it difficult to evaluate on MRI.^4^ Our study investigates the efficacy of a biparametric MRI (bpMRI)‐based deep learning algorithm for post‐FT PCa identification.

This retrospective cohort study utilized post‐FT prostate bpMRIs from an IRB‐approved clinical trial (NCT03354416). MRIs were evaluated with a previously developed AI model, a 3D U‐Net‐based deep neural network that can detect suspicious lesions on untreated prostate bpMRIs based on T2‐weighted images, apparent diffusion coefficient maps and high b‐value diffusion‐weighted images (Figure 1A–C).^5^ This algorithm was originally trained using a diverse MRI dataset obtained from treatment‐naïve patients.

**FIGURE 1:**
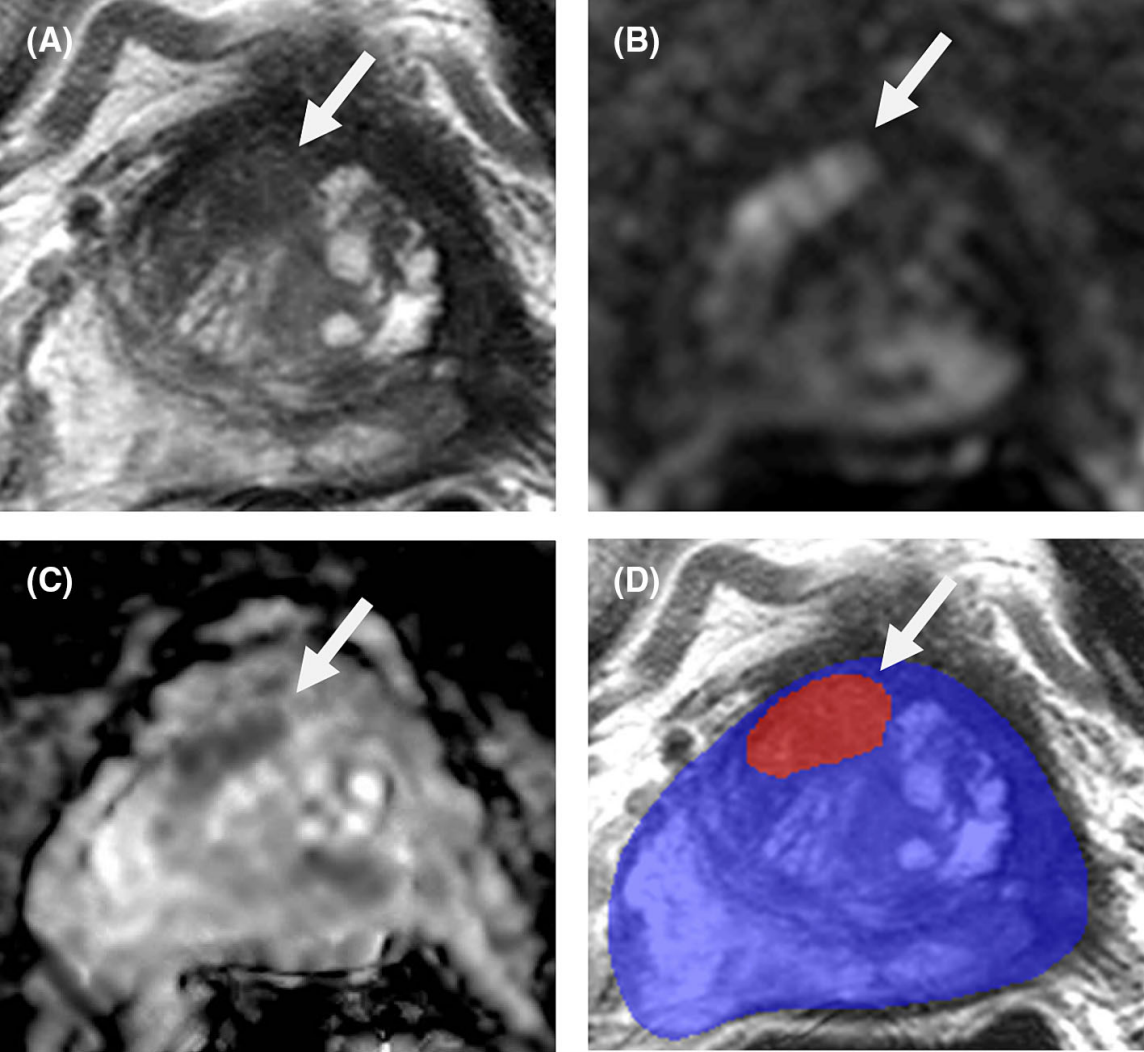
Biparametric MRI (bpMRI) of a 75‐year‐old patient post‐cryoablation with a serum PSA of 3.68 ng/ml. Axial MRI shows a homogenously hypointense lesion in the right mid anterior transition zone (arrow) on T2‐weighted MRI (A), hyperintensity on high b‐value (b = 1500 s/mm^2^) diffusion‐weighted MRI (B), hypointensity on apparent diffusion coefficient map (C), and binary lesion prediction model overlay on T2‐weighted image, with suspicious lesion predicted in red (D). MRI/TRUS fusion guided biopsy of this lesion demonstrated Gleason Grade Group 2 prostate cancer.

AI output consisted of PCa‐suspicious lesion prediction maps overlayed on T2‐weighted MRI (Figure 1D). Predictions were compared to MRI/transrectal ultrasound fusion‐guided and systematic prostate biopsies. A patient‐level analysis was performed where if at least one location containing Gleason Grade ≥1 disease was detected by the AI, this was a true positive. If an AI prediction was made in an area that turned out to be benign on biopsy, this was a false positive, even if biopsy revealed malignancy in a different region of the prostate. Patients with biopsy‐proven PCa lesions that were not predicted by AI were false negatives. If AI made no predictions in a patient with a fully benign prostate biopsy, this was a true negative. AI performance metrics included sensitivity, specificity, positive predictive value (PPV), negative predictive value (NPV) and overall accuracy.

Of the 40 included patients, the median time to post‐FT MRI was 2.5 years, and 25 patients had PCa at biopsy. AI made 33 unique lesion predictions across 24 patients. Of these, 16 patients (67%) had one lesion prediction, 7 patients (29%) had two lesion predictions and 1 patient (4%) had three lesion predictions. Across all AI predictions in this cohort, 9 patients (22.5%) had true positives, 15 patients (37.5%) had false positives, 10 patients (25%) had false negatives and 6 patients (15%) had true negatives. The AI's overall sensitivity was 47.4% with a specificity of 28.6%. The PPV and NPV were both 37.5%. Overall, the AI achieved an accuracy of 37.5%. The performance characteristics of this model are listed in Table 1.

**TABLE 1:** Performance metrics for AI performance in PCa lesion detection.

	TP	FP	FN	TN	Sensitivity	Specificity	PPV	NPV	Accuracy
PCa	9	15	10	6	47.4% (9/19)	28.6% (6/21)	37.5% (9/24)	37.5% (6/16)	37.5% (15/40)

Abbreviations: FN, false negatives; FP, false positives; NPV, negative predictive value; PCa, prostate cancer; PPV, positive predictive value; TN, true negatives; TP, true positives.

Our AI reached a moderate level of sensitivity. Despite low specificity and overall accuracy, this is a noteworthy finding, as this AI algorithm was trained on treatment‐naïve glands and not on post‐FT images. The 47% sensitivity rate underscores its potential and future effectiveness if specific training with post‐FT images could be achieved. This compares favourably to radiologist interpretations of MRI post‐FT, with some series demonstrating sub‐50% sensitivity.^6^, ^7^ Additionally, post‐FT MRI analysis typically relies on dynamic contrast‐enhanced (DCE) imaging over typical bpMRI sequences.^8^ However, our AI is based on bpMRI and does not include DCE MRI, so additional training on DCE data would likely require a complete renovation of the AI model.

Besides reliance on bpMRI, another limitation was the use of targeted and systematic prostate biopsies as the ground truth. While having whole gland specimens could have demonstrated whether lesions detected only by the AI were true or false positives, this may have resulted in a selection bias in our study population, as not all patients undergo surgery. Additionally, targeted biopsies were performed based on original prospective MRI read‐outs and not AI predictions. A standard‐of‐care system for radiologist analysis of post‐FT images has yet to be established, although the PI‐FAB system shows promise.^8^ Future AI algorithms will merit comparison to such standardized systems of interpretation.

In conclusion, the performance of this model in the post‐FT setting is noteworthy given the limitations of its training data and may already perform similarly to radiologist reads, although further research is necessary. This study provides motivation to improve the performance of a general AI model for prostate cancer lesion detection and serves as an initial step in understanding the potential role of AI in PCa detection in post‐FT patients.

## CONFLICT OF INTEREST

The authors declare no conflict of interest.
